# Editorial: Non-coding RNA in improving the efficacy of cancer immunotherapy

**DOI:** 10.3389/fimmu.2023.1210235

**Published:** 2023-05-09

**Authors:** Peixin Dong, Fabrizio Mattei, Mark Andrew Lindsay

**Affiliations:** ^1^ Department of Obstetrics and Gynecology, Hokkaido University School of Medicine, Hokkaido University, Sapporo, Japan; ^2^ Department of Oncology and Molecular Medicine, Istituto Superiore di Sanità, Rome, Italy; ^3^ Department of Life Sciences, University of Bath, Bath, United Kingdom

**Keywords:** non-coding RNA, microRNA, long non-coding RNA, cancer immunity, immunotherapy, tumor microenvironment

While the majority of the human genome is made up of non-coding RNAs (ncRNAs), which do not encode proteins, they may be divided into several types: circular RNAs (circRNAs), long non-coding RNAs (lncRNAs), and microRNAs (miRNAs). By modulating the translation of coding genes, these ncRNAs have a substantial influence on tumor biology. Furthermore, since ncRNAs are produced in a variety of immune cell types and may affect both innate and adaptive immunity, they are a prospective target for increasing cancer immunotherapy effectiveness.

Immune checkpoint inhibitor (ICI) treatments have limited therapeutic responses due to primary or acquired resistance, potentially driven by changes in the tumor microenvironment. Cancer cells can evade immune surveillance through the acquisition of epithelial-mesenchymal transition (EMT) phenotype and cancer stem-like cells (CSCs). MiRNAs have been studied for regulating CSC self-renewal and some miRNA-targeting therapies have entered clinical development for cancer treatment. However, recent studies suggest that lncRNAs also play critical roles in shaping the immunosuppressive tumor microenvironment (TME) and promoting tumor immune evasion, making them potential targets for enhancing anti-tumor immunity.

In this Research Topic, we aim to explore the regulatory roles of lncRNAs in the TME and cancer cells, their impact on tumor immune escape, and their potential as therapeutic targets for improving the efficacy of cancer immunotherapy.

Yan et al. investigated particular lncRNAs that affect important immune-related genes in non-small cell lung cancer, namely adenocarcinoma (AD) and squamous cell carcinoma (SCC). The research verified the prognostic importance of these lncRNAs in their cohorts by using risk models based on lncRNAs. The research discovered that individuals in the AD high-risk group and the SCC low-risk group had reduced immune responses, with dendritic cells playing a particularly crucial role in AD immune infiltration. Furthermore, the research predicted ICI therapy responses using T Cell Immune Dysfunction and Exclusion and immunophenoscore models, suggesting potential advantages for high-risk SCC patients. Finally, utilizing The Cancer Proteome Atlas and RNA-seq investigations of a transfected lung cancer cell line, the researchers identified the lncRNA LINC00996 as a possible therapeutic target in AD.

Lv et al. explored the effect of aberrant glycosylation, a protein alteration, in the development and progression of breast cancer (BC). The researchers found glycosyltransferase-related lncRNAs (GT-lncRNAs) linked with BC prognosis and responsiveness to immune checkpoint inhibitors (ICIs) therapy using co-expression analysis. They created a risk score based on eight GT-lncRNAs to predict BC patient outcomes and stratify patients based on immune infiltration. The risk score was shown to be a stronger predictor of BC prognosis than previously published models, and patients in the high-risk category had shorter survival. Furthermore, the risk score may aid in identifying individuals who might benefit the most from ICI therapy. Finally, the researchers provided experimental evidence that the lncRNA MIR4435-2HG may have a role in BC development through a variety of processes. The research indicated that prognostic markers based on GT-lncRNA might be useful tools for predicting BC outcomes and guiding treatment choices for personalized ICI-based immunotherapies.

Jiang et al. discussed the potential of ncRNAs in regulating immunological checkpoints in cancer therapy. Immune checkpoints such as PD-1, PD-L1, CD47, and BTLA are complexly regulated by ncRNAs, particularly miRNAs which bind to their 3’-untranslated region. CircRNAs also play a role in regulating immune checkpoints as “miRNA sponges.” There is still much to be learned about the specific roles of circRNAs and lncRNAs in regulating immune checkpoints, suggesting a promising avenue for further research. Nonetheless, the precise targeting ability of ncRNAs for immune checkpoints shows potential for developing innovative and effective immunotherapies for cancer.

Jia et al. provided a summary of recent research on the functions and mechanisms of exosomal ncRNAs in the TME. Exosomes, which carry regulatory molecules, proteins, and nucleic acids, are important in cellular communication within the TME. The article specifically highlights the role of exosomal ncRNAs, such as lncRNAs, miRNAs, and circRNAs, in regulating TME function and contributing to cancer development and progression. This review emphasizes the central role of exosomes in remotely transporting RNAs and shaping the TME.

The article by Wu et al. summarized the mechanisms by which circRNAs affect the TME in digestive system cancers, such as immune surveillance, angiogenesis, EMT, and extracellular matrix remodeling. This review also discussed how specific regions of several circRNAs can impact three key events of the TME, namely autophagy, apoptosis and proteins involved in drug efflux control. In this view, the regulation of the TME by circRNAs is considered as a potential new therapeutic approach for cancer.

Zhang et al. discussed the processes by which pyroptosis-associated ncRNAs trigger pyroptosis, their impact on tumor resistance and the tumor microenvironment, and prospective biomarkers and therapeutic targets. NcRNA-regulated pyroptosis may improve tumor resistance while simultaneously inducing a significant inflammatory response and remodeling the tumor microenvironment. This study proposed that targeting pyroptosis-related ncRNAs might be a feasible therapeutic method to reduce tumor resistance, and those pyroptosis-related ncRNAs could act as biomarkers for diagnosis and immunotherapy success.

Zhou et al. debated on the role of lncRNAs as key modulators of cells resident inside the melanoma tumor microenvironment. In particular, they evidence that several oncogenic and tumor suppressor lncRNAs are endowed with the ability to directly modulate the melanoma immune environment by controlling different immune cell subsets infiltrating the TME. These features can be potentially exploited to specifically target the TME *via* lncRNA-based immunotherapeutic strategies.

In conclusion, ncRNAs regulate coding genes and modulate immunity, making them promising targets for enhancing cancer immunotherapy. Specific lncRNAs have been identified as novel targets for cancer therapy. NcRNAs also regulate immunological checkpoints, suggesting the potential for developing effective immunotherapies for cancer. Exosomal ncRNAs play a role in regulating TME function and further investigation will provide valuable insights into cancer immunity regulation. Targeting ncRNAs, including miRNAs, lncRNAs and circRNAs, is a potential strategy to overcome immunoresistance and enhance tumor immunogenicity.

## Author contributions

All authors listed have made a substantial, direct, and intellectual contribution to the work and approved it for publication.

